# A comparison between Qigong exercise and cycle ergometer exercise for the rehabilitation of chronic obstructive pulmonary disease: A pilot randomized controlled trial (CONSORT)

**DOI:** 10.1097/MD.0000000000026010

**Published:** 2021-05-28

**Authors:** Xiaosheng Dong, Xiangyu Wang, Ningxin Jia, Xianhai Chen, Meng Ding

**Affiliations:** a College of Physical Education, Shandong Normal University; b College of Physical Education, Shandong University, Jinan; c Capital Institute of Physical Education, Beijing; d Affiliated Hospital of Shandong, University of Traditional Chinese Medicine, Jinan, China.

**Keywords:** chronic obstructive pulmonary disease, Qigong exercise, cycle ergometer exercise, quality of life, cardiopulmonary endurance, severity of clinical symptoms

## Abstract

**Background:**

Chronic obstructive pulmonary disease (COPD) is a common respiratory disease that is associated with significant morbidity and mortality. Exercise training confers health benefits to people with COPD. The purpose of this study was to compare differences in the rehabilitation of COPD between Qigong exercise (QE) and aerobic exercise using a cycle ergometer (CE).

**Methods:**

This study was a randomized single-blind controlled trial. Twenty six participants were recruited and randomized to either the Qigong group or the cycle ergometer group. Both interventions lasted 12 weeks and comprised a 30 minutes supervised training session performed twice a week, that is, 24 sessions in total. The primary outcome measure was the endurance capacity measured by the six-minute walk test (6MWT). The secondary outcome measures were the results of the St. George's Hospital Respiratory Questionnaire (SGRQ) and the COPD assessment test (CAT).

**Results:**

Participants in the group that performed aerobic exercise using a cycle ergometer had significantly improved 6MWT (*P* = .005), SGRQ (*P* = .029), and CAT (*P* = .018) results. Participants in the Qigong exercise group had significant changes in 6MWT (*P* = .033). However, the differences in 6MWT and SGRQ were not statistically significant between the 2 groups. The changes in CAT scores before and after the intervention were significantly different between the 2 groups (*P* = .020). There were no reports of adverse events during the course of the trial.

**Conclusions:**

There was no difference in the primary outcome between groups. In particular, QE and cycle ergometer exercise had similar rehabilitation effects on the improvement of the cardiopulmonary endurance and quality of life of chronic obstructive pulmonary disease patients. In addition, cycle ergometer exercise may lead to a better trend of improvement in the quality of life and can improve the severity of the clinical symptoms of chronic obstructive pulmonary disease.

**Trial registration:**

ChiCTR-TRC-14004404.

## 1 Introduction

Chronic obstructive pulmonary disease (COPD), a common respiratory disease characterized by persistent airflow limitation, is a leading cause of morbidity and mortality worldwide.^[1]^ A cross-sectional survey in China between 2002 and 2004 demonstrated a high prevalence of COPD in middle-aged and elderly people.^[2]^ A study indicated that 8.6% of the general Chinese adult population aged 20 years or older in 2015 had spirometry-defined COPD, which has reached epidemic proportions, having become significantly higher than the estimates reported by the Global Burden of Diseases.^[3]^ Patients with COPD are involved in a vicious cycle of inactivity, initiated by breathlessness that occurs with physical activity.^[4,5]^ Pharmacological therapy leads to improvements in breathing but has a limited effect on physical deconditioning. Pulmonary rehabilitation serves as an essential component of the management of COPD and is beneficial for improving health-related quality of life and exercise capacity; it relieves dyspnea and fatigue, improves emotional function, and enhances the sense of control that individuals have over their condition.^[6]^ Exercise training, an important part of pulmonary rehabilitation, has been shown to improve dyspnea and health status and to decrease health care use in patients with COPD.^[4]^

Qigong, an ancient Chinese exercise, involves movement of the extremities, meditation, and breathing control. It is used to promote a healthy lifestyle and to treat various chronic diseases.^[7–10]^ Recently, the use of Qigong was recommended for COPD rehabilitation, and some clinical trials have been conducted to evaluate the effects of Qigong on patients with COPD.^[11,12]^ According to the results of our prior systematic review, Qigong intervention demonstrates more benefits for exercise tolerance quality of life (QoL) and dyspnea remission than interventions without exercise; however, it was not been confirmed whether Qigong is a better complementary treatment for physical activity and QoL in COPD rehabilitation compared with other exercise training forms such as walking.^[13]^

Some randomized controlled trials have investigated the effects of Qigong exercise (QE) on the rehabilitation of patients with COPD.^[14–17]^ However, most of the studies compared Qigong with no exercise^[16,17]^ or an aerobic exercise such as walking.^[14–17]^ In addition, the cycle ergometer training has been shown to improve exercise performance, dyspnea, and quality of life in patients with COPD, and therefore has been accepted as an important part of modern Western pulmonary rehabilitation.^[18]^ There are few studies comparing QE with modern Western exercise rehabilitation methods in patients with COPD. There is a need to identify the exercise modalities that have better rehabilitative effects on COPD patients so as to deliver more meaningful for individualized pulmonary rehabilitation in the future. Therefore, the aim of the present study was to compare the differences in the rehabilitation of COPD between the QE and the cycle ergometer exercise (CE).

## 2 Methods

This randomized controlled trail (RCT) study was approved was approved by the ethic committee of the Affiliated Hospital of Shandong University of Traditional Chinese Medicine. This study was registered in the Chinese Clinical Trial Registry, which is in the WHO Registry Network (registration number: ChiCTR-TRC-14004404).

### 2.1 Study design

This study was a parallel-group, randomized controlled trial to compare QE with CE in patients with a stable stage of COPD after a 12-week intervention.

### 2.2 Participants

Twenty six participants were recruited from the department of lung disease of the Affiliated Hospital of Shandong University of Traditional Chinese Medicine, Jinan, China. The inclusion criteria were:

1. ambulatory male and female,
2. aged between 40 and 75 years with COPD within GOLD stages I–III.^[1]^

Exclusion criteria were

1. age >75 years,
2. acute exacerbation of COPD within the last 4 weeks,
3. cancer,
4. asthma,
5. bronchiectasis,
6. symptomatic cardiovascular disease, or
7. other systemic or musculoskeletal diseases that can hinder exercise training.

All participants provided written consent before participation in the study.

### 2.3 Intervention

Both interventions lasted 12 weeks and comprised a 30 minutes supervised training session, performed twice a week, that is, 24 sessions in total.

### 2.4 Qigong intervention

**Figure 1:**
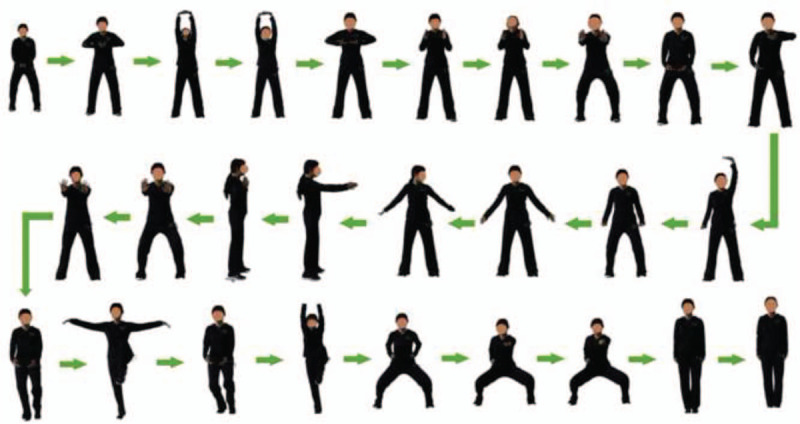
The Qigong exercise form.

QE was taught by an instructor with long-term QE teaching experience. Patients learned and mastered the QE form in the first 2 weeks. From the third week, the intensity of the QE training was monitored by heart rates telemetry and the level of exertion. To increase (or maintain) the intensity to a moderate level, patients were asked to wave their upper limbs more vigorously or to imagine pushing against resistance during movements. Natural breathing was recommended at the beginning. The patients achieved the proper combination of movements, breathing, and mindfulness with the constant practice (Fig. 1)

### 2.5 Cycle ergometer intervention

Patients in the CE group performed the cycle ergometer exercise at ≥60% of peak work rates.^[14]^ The peak work rates were calculated by Luxton equation using the results of the six-minute walk distance (6MWD).^[19]^ The intensity of cycle ergometer exercise was gradually increased to the target intensity in the first 2 weeks. We modified the intensity depending on the patient's response, heart rates, or SPO2 during the exercise. If the patients felt very breathless or severe leg fatigue, or had high heart rates (>80% peak heart rate) or low SPO2 (<90%), we lowered the work rate.

### 2.6 Outcome measures

All outcome measures were recorded at baseline and at the end of the study period.

The primary outcome was the endurance capacity measured by the 6MWT,^[15–17,20]^ in which participants walked up and down a 100 ft (30-m) hallway for 6 minutes after receiving instructions to cover as much distances as possible. The distance completed after 6 minutes was recorded. The 6MWT has acceptable test–retest reliability and divergent validity in patients with COPD.^[21]^ The secondary outcomes were the results of 2 self-reported questionnaires (Chinese version): The St. George's Hospital Respiratory Questionnaire (SGRQ)^[22–24]^ and the COPD assessment test (CAT).^[25]^ The SGRQ^[26,27]^ and CAT^[28,29]^ have acceptable test–retest reliability and divergent validity in patients with COPD

### 2.7 Randomization allocation

The patients admitted to the study were randomized to either the QE group or the CE group. The allocation sequence was generated through a random table in excel by a designer who was not involved in assessing participants.

### 2.8 Blinding procedure

Allocation was undisclosed by the study designer until the end of the study to ensure allocation concealment. Outcome assessors and data analysts remained blinded to the allocation.

### 2.9 Statistical analysis

The study results underwent statistical analysis with SPSS software version 21.0 (SPSS Inc., Chicago, IL). Descriptive statistics were performed on all variables by *t* test according to gender. Continuous variables are expressed as the mean and standard deviation (mean ± standard deviation). Effect sizes (Cohen *d*) were categorized as large if *d* > 0.8, medium if *d* > 0.5, and small if *d* > 0.2.^[30]^ A covariance analysis was adopted for comparisons between groups, and a paired *t* test was used for before-exercise and after-exercise comparisons within a group, whereas *P* < .05 represents a significant difference between the test results.

### 2.10 Patient and public involvement

No participants were involved in setting the research question or in the design or conduct of this study. No participants were asked to advice on the interpretation or writing up of the results. There are no plans to disseminate the results of the research individually to study participants.

## 3 Results

### 3.1 Participants

**Figure 2:**
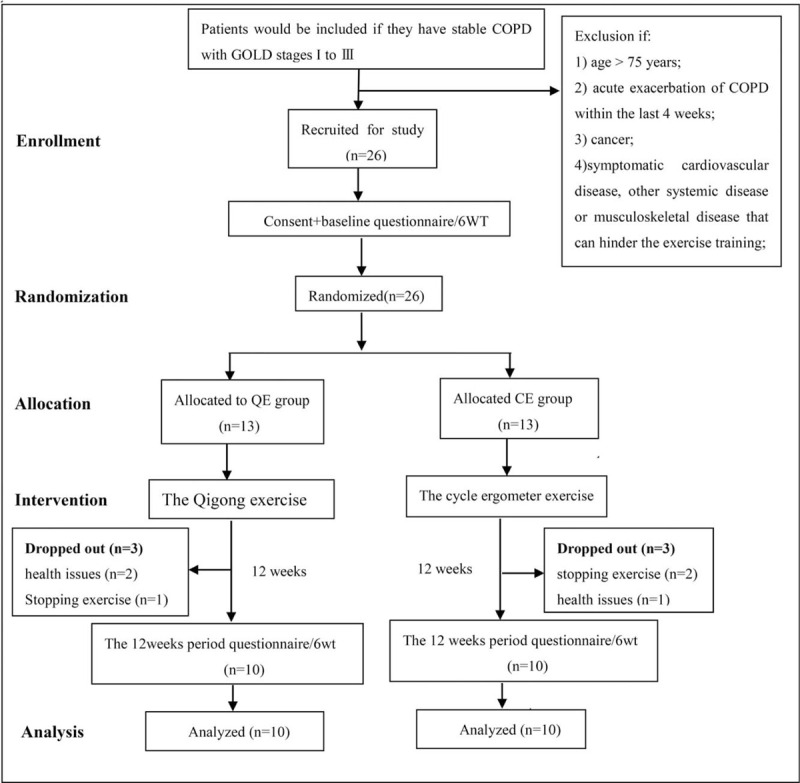
The flow chart of the trial design.

Twenty six participants were randomly assigned to the groups: The QE group (n = 13; male = 9, female = 4) and the CE group (n = 13; male = 10, female = 3). Over the course of the study, 3 participants in the QE group withdrew because they experienced health issues (n = 2) or stopped the exercise (n = 1); 3 participants in the CE group withdrew because they stopped the exercise (n = 2) or had health issues (n = 1). The data from the 20 participants who completed the study were included in the final analysis. Figure 2 shows the flow of subjects following the recommendation of the Consolidated Standards of Reporting Trials (CONSORT).^[31]^Table 1 shows the data on subject characteristics. No differences in the values of age, weight, or height were found between the groups using one-way ANOVA.

**Table 1:** Subject characteristics for each group. Data are means (±SD).

Demographic	QE group (n = 10)	CE group (n = 10)	*P* value
Age (years)	65.50 (6.26)	63.60 (7.88)	.558
Weight (kg)	64.60 (11.39)	71.10 (11.81)	.538
Height (m)	1.68 (0.08)	1.70 (0.06)	.226

### 3.2 6MWT

Table 2 shows the mean 6MWD of the 2 groups at baseline and end-of-study measurements that we had scheduled. There were no statistically significant differences between the 2 groups (*P* = .694). However, the changes in 6MWD in the 2 groups showed a similar tendency. The mean values of 6MWD of the 2 groups increased at the end of the study, and the differences were significant (*P* ≤ .05; effect size (QE) = 0.45; effect size (CE) = 0.92).

**Table 2:** The means (±SD) of 6MWT, SGRQ, and CAT in 2 groups.

Variables	QE group (n = 10)		QE groupgroup paired *t* test (P)	QE group effect Size (95% CI)	CE group (n = 10)		CE group paired *t* test(P)	CE group effect size (95% CI)	Baseline ANOVA analysis (P)	ANCOV aanalysis (P)
	Baseline	12 Weeks			Baseline	12 Weeks				
6MWT	530.00 (75.38)	559.80 (55.8)	.033	0.45 (−0.45,1.33)	520.60 (35.55)	549.60 (26.94)	.005	0.92 (−0.02,1.83)	.727	.694
SGRQ	27.82 (11.82)	22.48 (8.87)	.210	−0.51 (−1.40,0.39)	34.43 (13.00)	23.41 (10.40)	.029	−0.94 (−1.85,0.01)	.250	.862
CAT	17.00 (3.16)	19.10 (3.60)	.155	0.62 (-0.29,1.51)	20.10 (5.57)	16.50 (3.27)	.018	-0.79 (-1.69,0.14)	.143	.020

### 3.3 SGRQ

The changes in the SGRQ score in the 2 groups showed a similar tendency (see Table 2). The results in the QE and CE groups had a degree of decline after the interventions. The difference in the CE group was significant (*P* = .029; effect size = 0.94), but there was no significant difference in the QE group (*P* = .210; effect size = 0.51). There was no significant difference between the 2 groups (*P* = .862).

### 3.4 CAT

Compared with before the intervention, the CAT score of the QE group increased after the intervention, but the difference was not significant (*P* = .155; effect size = 0.62). The CE group showed a decrease in the CAT score, and the difference was significant (*P* = .018; effect size = 0.79). There were statistically significant differences between the 2 groups (*P* = .020). The specific results and statistical analysis are shown in Table 2.

## 4 Discussion

In the present study, we compared the effects between the QE and cycle ergometer exercise on the rehabilitation of patients with COPD. We determined that the group that performed aerobic exercise using a cycle ergometer had statistically significant improvements in their 6MWT, SGRQ, and CAT results 12 weeks after the intervention compared with the baseline. The 6MWD in the Qigong group had a statistically significant improvement. However, SGRQ and CAT were not significantly improved in the Qigong group. There were no statistically significant differences in 6MWT and SGRQ between the 2 groups, which were associated with improvements in cardiorespiratory capacity and QoL. However, the difference between the Qigong and cycle ergometer exercise groups reached statistical significance only for CAT, suggesting a reduction in disease severity.

According to our review of the literature, several studies estimating the effects of Qigong on COPD have indicated that Qigong should benefit patients with COPD.^[12,13,25,32–43]^ However, most of the previous studies on Qigong for COPD were of low methodological quality.^[13,36]^ Moreover, it cannot be confirmed that Qigong is a better complementary treatment in COPD rehabilitation compared with other exercise training forms. Many previous studies have compared Qigong to walking, breathing control, routine daily life, and usual care,^[25,33–35,39,44]^ but few have compared it with aerobic exercise using a cycle ergometer. A previous study^[37]^ showed that Tai Chi produces a physiological response similar to that elicited by exercise on a constant-rate treadmill at 60% of maximum load. In this study, we confirmed that 12 weeks of either Qigong or cycle ergometer training should improve the endurance capacity and quality of life of COPD patients.

The 6MWT has been used as a simple, reliable, and valid assessment of exercise tolerance in COPD.^[15,16]^ The 6MWD is a better predictor of physical activity and mortality in patients with COPD than other methods of assessment. After 12 weeks of exercise intervention, the 6MWD of the QE and CE groups increased, and the difference was significant. However, there was no significant difference between the groups. This shows that QE and aerobic exercise using a cycle ergometer can improve the physical activity level and cardiopulmonary endurance and can lower the mortality of COPD patients. However, the difference in the CE group was not substantial enough and needs to be confirmed by further study. This result is similar to that of previous studies^[13,25,33,35,38–40,42,44,45]^ that confirmed that Qigong can improve the cardiorespiratory function of COPD patients.

The SGRQ is a questionnaire on the self-reported symptoms and quality of life in patients with diseases of the airways (both asthma and chronic obstructive airway disease). Previous research has demonstrated that the SGRQ is a valid, repeatable, and sensitive measure of the health status of patients with COPD. The SGRQ comprises 3 dimensions (symptoms, impact, and activity) and a global score. Each dimension receives a score between 0 and 100, with 0 representing a complete lack of deterioration.^[22–24]^ After 12 weeks of the exercise intervention, although there were no statistically significant differences between the 2groups, the results for the 2 groups had decreased, although the difference in the QE group was not significant. However, the difference was significant in the CE group. These results show that the quality of life in the 2 groups follows an improving trend.

The CAT score of the CE group was significantly reduced, and the difference between the scores before and after the test was significant. There was a significant difference between the 2 groups when comparing their changes. This suggests that aerobic exercise using a cycle ergometer can reduce the severity of clinical symptoms (based on the CAT score) of COPD patients. In our study, QE had no effect on improving the CAT score in COPD patients. However, a previous study^[25]^ confirmed that Yi Jinjing, a form of QE, can improve the basic consistency of CAT in patients with COPD. The ineffectiveness in improving the CAT score in our study may be related to the short time of the intervention, the relatively low COPD level of the subjects at the beginning of the study, the need to learn QE for a period of time, and unskilled movement, but the intensity of the aerobic exercise could be well controlled.

In the present study, although QE and cycle ergometer exercise both had a rehabilitative effect on cardiopulmonary endurance and quality of life in COPD patients, cycle ergometer exercise had advantages in reducing the severity of clinical symptoms of COPD. The 2 exercise forms have different characteristics. Quantification and standardization are the main features and advantages of CE; however, the mechanical exercise program and monotonous exercise style often make patients feel tired. Qigong, as a traditional exercise belonging to Chinese Traditional Medicine, is not as accurate as exercise that is typically used in Western medicine in terms of the prevention and treatment of chronic disease. However, Qigong emphasizes personal understanding and cultural identification of practitioners, and as an exercise with a combination of movement and meditation, it could suppress sympathetic activation.^[46]^ In addition, Qigong can increase the strength of the respiratory muscles, reduce the pulmonary residual volume, promote efficiency in gas exchange, and slow the decrease in lung function.^[47]^ Therefore, using a combination of Qigong and modern exercise in the rehabilitation of COPD could perhaps have greater efficacy than using a single exercise form. This should be determined in a future study.

As a pilot study, there are several limitations in the present research. First, the sample size of the present study was not large enough, and therefore, it is difficult to generalize the results to a whole population of COPD patients. Further studies should expand the sample size to make the effects generalizable. Second, there was no negative control group (no exercise) in this study. Third, the duration of our trial intervention was not long enough.

## 5 Conclusion

According to the results of this study, there was no difference in the primary outcome between groups. In particular, QE and cycle ergometer exercise had similar rehabilitation effects on improving the cardiopulmonary endurance and quality of life in COPD patients. In addition, cycle ergometer exercise may have a better trend of improvement in the quality of life and can improve the severity of clinical symptoms of COPD. However, the results need to be cautiously interpreted and should be confirmed in further clinical trials because of the pilot character of this study. A large sample size trial is needed to compare the effects of QE and CE on COPD in the future.

## Acknowledgments

We want to express our gratitude to the China Postdoctoral Science Foundation and health care Qigong project of Chinese General Administration of Sport for making this study possible.

## Author contributions

**Conceptualization:** Xiaosheng Dong, Meng Ding.

**Data curation:** Xiangyu Wang, Ningxin Jia.

**Investigation:** Xiaosheng Dong, Xiangyu Wang.

**Methodology:** Xiaosheng Dong, Meng Ding.

**Writing – original draft:** Xiaosheng Dong.

**Writing – review & editing:** Xianhai Chen, Meng Ding.
